# Astrocyte development in the cerebral cortex: Complexity of their origin, genesis, and maturation

**DOI:** 10.3389/fnins.2022.916055

**Published:** 2022-09-13

**Authors:** Solène Clavreul, Laura Dumas, Karine Loulier

**Affiliations:** Institute for Neurosciences of Montpellier (INM), Univ Montpellier, INSERM, Montpellier, France

**Keywords:** astrocytes, cerebral cortex, gliogenesis, proliferation, maturation

## Abstract

In the mammalian brain, astrocytes form a heterogeneous population at the morphological, molecular, functional, intra-, and inter-region levels. In the past, a few types of astrocytes have been first described based on their morphology and, thereafter, according to limited key molecular markers. With the advent of bulk and single-cell transcriptomics, the diversity of astrocytes is now progressively deciphered and its extent better appreciated. However, the origin of this diversity remains unresolved, even though many recent studies unraveled the specificities of astroglial development at both population and individual cell levels, particularly in the cerebral cortex. Despite the lack of specific markers for each astrocyte subtype, a better understanding of the cellular and molecular events underlying cortical astrocyte diversity is nevertheless within our reach thanks to the development of intersectional lineage tracing, microdissection, spatial mapping, and single-cell transcriptomic tools. Here we present a brief overview describing recent findings on the genesis and maturation of astrocytes and their key regulators during cerebral cortex development. All these studies have considerably advanced our knowledge of cortical astrogliogenesis, which relies on a more complex mode of development than their neuronal counterparts, that undeniably impact astrocyte diversity in the cerebral cortex.

## Introduction

Astrocytes are key cellular partners of neurons and blood vessels in the central nervous system. The last two decades have seen an accumulation of new studies aiming at characterizing these cells initially considered as simple support cells for neurons. All these works have progressively revealed an unexpected diversity of these astrocytes in the brain where they constitute a heterogeneous population at the morphological, molecular, functional, inter-, and intra-region levels (Khakh and Deneen, 2019). Various astrocyte subtypes have been first described based on their morphology and few key molecular markers such as GFAP for white matter fibrous and reactive astrocytes and S100ß for gray matter protoplasmic astrocytes. Nowadays, additional molecular markers for cortical astrocytes have been described, such as NFIA, GLAST, Sox9, or Aldh1l1, enabling the investigation of astrocyte physiology (Molofsky et al., 2012). Until now, the cellular and molecular mechanisms underlying the establishment of astrocyte diversity during development have remained difficult to explore due to the absence of specific markers for each astrocyte subtype. In recent years, refinements in cell lineage tracking techniques that have moved to multicolor to increase the number of clones that could be tracked simultaneously, reviewed in Dumas et al. (2022), and in high-throughput transcriptomics (Wagner and Klein, 2020) have elucidated key elements of cortical astrocyte development in the mammalian brain. In this minireview, we compile in synthetic form the latest findings on the development of cortical astrocytes from their multiple sources of production to the key factors regulating their generation and maturation which together highlight the complexity of the genesis of cortical astrocytes.

## Origins of cortical astrocytes

### Embryonic source

#### Radial glia

**FIGURE 1:**
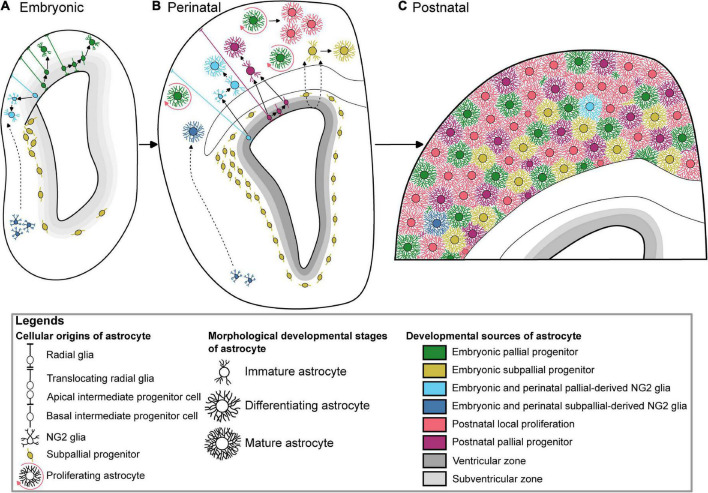
Multiple developmental origins underlying the generation of cortical astrocytes. **(A)** Embryonic astrogliogenesis relies on immature astrocytes deriving from translocating pallial radial glia, multipotent intermediate pallial progenitors, two independent oligodendrocyte progenitor cells sources, and subpallial progenitors. **(B)** New cortical perinatal astrocytes are generated from radial glia, local proliferation, intermediate progenitors, pallial and subpallial oligodendrocyte progenitor cells, and colonizing subpallial progenitors. **(C)** Color-coded protoplasmic astrocytes based on their respective developmental sources tiling the whole postnatal cortex. The postnatal local proliferation is mainly observed in the upper layers, while subpallial-derived astrocytes are GFAP-positive suggesting their lower layer localization. Of note, these subpallial-derived astrocytes have not been described after P10. The dotted arrows show hypothetic migration paths.

At the end of the neurogenic phase around the 16th embryonic day (E16) during mouse development, radial glial cells (RGC) lose their neurogenic potential and progressively acquire most of astrocyte features (Mori et al., 2005). This gliogenic switch is regulated by intrinsic and epigenetic factors (Adnani et al., 2018). Radial glia produces most of astrocyte precursors by E18, which subsequently migrate to the white or grey matter where they differentiate into fibrous and protoplasmic astrocytes, respectively (Tabata, 2015). Cell lineage studies have revealed the existence of bipotent progenitors successively producing neurons and astrocytes, as well as restricted progenitors generating only certain neuronal or glial subtypes (Kriegstein and Alvarez-Buylla, 2009). Mosaic Analysis with Double Markers (MADM) clonal analysis show that 1/6 of neurogenic radial glia cortical progenitors produce glia (Gao et al., 2014). Remaining RGC eventually differentiate directly into astrocytes (Figure 1). They undergo morphological changes, lose their apical contact, become unipolar and retract their radial fibers, before moving away their cell body from the ventricular zone and becoming multipolar, thus acquiring their astrocyte morphology (Kriegstein and Alvarez-Buylla, 2009). In addition to direct transformation into astrocyte precursors, RGC generate apical multipotent intermediate progenitors that express ASCL1 and EGFR as revealed by Li and collaborators using a combination of single-cell RNA-Seq with intersectional lineage analysis (Li et al., 2021). The colonization of the neocortical wall is achieved by a fraction of apical cortical progenitors which delaminate from the ventricular zone before birth (Figure 1). Multiplexed clonal analysis based on multicolor MAGIC Markers strategy reveal that astrocyte precursors disperse prenatally in a non-stereotyped way in the cortical parenchyma where they expand as scattered clonal units, which can result in a sparse distribution of sibling astrocytes at later stages (Clavreul et al., 2019; Ojalvo-Sanz and López-Mascaraque, 2021). Aside from RGC, cortical astrocytes arise from alternative embryonic sources that might contribute to their diversity. It includes oligodendrocyte progenitor cells, Olig2 progenitors and embryonic subpallial progenitors.

#### Oligodendrocyte progenitor cells

Oligodendrocyte progenitor cells (OPCs) are glial cells identified by their expression of NG2, Olig2, PDGFRα, or PLP markers. Cortical astrocyte subpopulations are generated from at least two regionally distinct sources of NG2-expressing OPCs: a ventral subset (Huang et al., 2014, 2019) and pallial progenitor derivatives (Sánchez-González et al., 2020; Figure 1). Their potential to differentiate into astrocytes is however transient from E12 (Huang et al., 2019) to neonatal stage (Huang et al., 2014; Sánchez-González et al., 2020). Unlike their multipotent dorsal counterpart, ventral OPCs appear to be monopotent glial progenitors (Zhu et al., 2011). In contrast, NG2-expressing OPCs deriving from pallial progenitors produce both protoplasmic and fibrous astrocytes (Sánchez-González et al., 2020). In the adult dorsal cortex, up to 1.6% of S100β + protoplasmic astrocytes arise from these two distinct sources of OPCs (Zhu et al., 2008). In addition to NG2-Cre (Zhu et al., 2008), NG2-Cre^ERTM^ BAC (Zhu et al., 2011), NG2-Cre^ERT2^ KI (Huang et al., 2014, 2019), the fate of OPC has been tracked using other mouse lines such as PLP-Cre^ERT2^ (Doerflinger et al., 2003; Guo et al., 2009, 2010), Olig2-Cre^ERTM^ (Takebayashi et al., 2002; Dimou et al., 2008), Pdgfrα-^Cre/ERT2^ (Rivers et al., 2008; Kang et al., 2010). Protoplasmic astrocytes can be generated from early PLP-positive OPCs while no astrocytes are observed after adult induction of the Cre recombinase in PLP-Cre^ERT2^; reporter mice confirming their transient potential to give astrocytes (Guo et al., 2009, 2010).

#### Olig2 progenitors

On the other hand, from 5 to 11% of reporter-positive astrocytes are found in the grey matter at 6 months after adult induction in Olig2Cre^ERTM^; reporter mice (Dimou et al., 2008). However, Olig2 expression is not restricted to NG2 glia as some embryonic and even adult subventricular zone (SVZ) neural progenitor cells also express this marker. Olig2 is a bHLH transcription factor involved in cortical astrocyte development, from the specification to differentiation stages. Indeed, most, if not all, protoplasmic astrocytes issued from cortical progenitors transitioned by the Olig2 lineage as clearly demonstrated by Olig2-Cre genetic fate mapping combined with multicolor reporters (Clavreul et al., 2019). It has been shown that Olig2 promotes macroglia identity by repressing the neuronal phenotype among certain neural progenitors arising from both the pallium and subpallium (Marshall et al., 2005). Moreover, Olig2 participates in the astrocyte differentiation in the dorsal pallium, but not in the basal forebrain (Ono et al., 2008). In the cerebral cortex, Olig2 is progressively downregulated as astrocytes mature (Cai et al., 2007; Zhu et al., 2012) while its expression is maintained in other regions. Indeed, Olig2 is a marker for more than 80% of the mature astrocytes located in the globus pallidus, olfactory bulb, midbrain, thalamus, medulla, and spinal cord (Tatsumi et al., 2018; Wang et al., 2021). In the spinal cord, Olig2 progenitors of the pMN domain give rise to motor neurons, OPCs as well as a subpopulation of Olig2+ astrocyte progenitors that retained Olig2 expression as they differentiate and mature until adulthood (Ohayon et al., 2019). Interestingly, Olig2+ and GFAP+ astrocytes occupy mutually exclusive areas in the adult brain (Tatsumi et al., 2018). In the globus pallidus, the Olig2-astrocyte subset tends to express GABA transporter-3 and/or SLC7A10 transporter of NAA (Tatsumi et al., 2018, 2021), suggesting that a molecularly and regionally distinct subpopulation of astrocytes may exert specific functions. In addition, Olig2-expressing astrocyte subtype is as well-found in the juvenile spinal cord and exhibits a unique gene expression signature that includes *inka2*, *kcnip3*, and *slc7a10* showing a first link between a developmental origin of an astrocyte subtype and its molecular identity that could influence synapse organization and activity (Ohayon et al., 2021). In contrast, cortical astrocytes derived from pallial and subpallial sources exhibit a perivascular shape that indicates common function between developmentally distinct populations (Marshall and Goldman, 2002). Adult astrocytes can act as quiescent neural stem cells. Indeed, mitotic and cell cycle control genes are expressed in a rare subpopulation of uniformly distributed putative astrocyte progenitors (AST5) and a specific hippocampal subset of neural stem cell (AST4) that could proliferate in response to pathological stimulus in the mouse (Batiuk et al., 2020). Interestingly, in the striatum and somatosensory cortex, parenchymal astrocytes have recently been shown to be latent neural stem cells, capable to generate neuroblasts upon treatment with stroke-related and mitogen cues (Magnusson et al., 2020).

#### Subpallial origin

In the forebrain and in the spinal cord, astrocytes are born from RGC within the boundaries of their allocated territories delineated by their neuroepithelial progenitors (Hochstim et al., 2008; Tsai et al., 2012). Nonetheless, few studies hints that some cortical astrocytes may arise from outside the cerebral cortex. An overlooked source of cortical astrocytes comes from subpallial progenitors located in the ganglionic eminences (Marshall and Goldman, 2002; Nery et al., 2002; Figure 1). These progenitors belong to the Dlx2 lineage reflecting their ventral telencephalic origin and they emigrate dorsally toward the cortical parenchyma. They colonize the core of the dorsolateral corner of the perinatal SVZ by progressively displacing Aldolase C/Zebrin-II + pallial resident progenitors to the edge (Staugaitis et al., 2001; Marshall and Goldman, 2002). Enrolled into the late wave of an uncommon medial tangential migration stream (E14–E16), Dlx2 + subpallial progenitors migrate within the dorsal periventricular zone (Anderson et al., 2001). Two subpallial astrocyte progenitor subtypes expressing either Sparc or Sparcl1 have recently been identified in the perinatal cortex suggesting that molecularly divergent astrocytes derived from the subpallium might differently regulate neuronal synaptic formation (Liu et al., 2022). *Aldh1l1* gene and Sparc expression pattern is gradually increased along the dorsoventral axis of the adult brain (Morel et al., 2017). The regulation of synaptic activity has been shown to be region-selective. Indeed, subcortical astrocytes appear less competent at modulating the function of cortical neurons *in vitro* (Morel et al., 2017). Subpallial Dlx2-expressing progenitors develop notably into GFAP-positive astrocytes and oligodendrocytes in the juvenile cortex, white matter and striatum (Marshall and Goldman, 2002; Nery et al., 2002). Likewise, Dlx2 is a key factor used to reprogram both adult astrocyte (Heinrich et al., 2010) and OPC (Boshans et al., 2021) into GABAergic interneuron or tripotent neural progenitor cell (Zhang et al., 2022). Interestingly, astrocytes originating from both pallial and subpallial progenitors have been recently shown to converge to a similar postnatal transcriptional signature by combining STICR barcoding and scRNA-seq (Bandler et al., 2022). However, the extent of the contribution of this subpallial subset to the cortical astrocyte population is unknown. It is also unclear if these ventrally-derived astrocytes survive beyond the postnatal day (P) 10 (Marshall and Goldman, 2002) and if they play a specific function in the cerebral cortex.

### Postnatal production

#### Postnatal subventricular zone progenitors

After birth, a loss of RGC occurs (Marshall et al., 2003). Astrocytes generated afterward are thought to be issued from SVZ progenitors, which are not, unlike RGC, attached to the pial surface (Figure 1). The postnatal contribution of SVZ progenitors to astrocyte production will continue until P14 (Levison and Goldman, 1993). While Nestin expression characterizes RGC, a study using Nestin-Cre^ER^ mice also showed proliferating Nestin + glial progenitors in the SVZ and detached from the pial surface, after tamoxifen injection at the end of embryogenesis (Burns et al., 2009). This confirms that perinatal gliogenesis occurs in both the VZ with a RGC origin, and the SVZ, where intermediate progenitors give birth to cells migrating to the white matter and to the cerebral cortex and differentiating into astrocytes and oligodendrocytes. However, the postnatal contribution of SVZ progenitors to astrocyte production has been challenged by a study using postnatal electroporation of episomal reporters to label postnatal SVZ progenitors which shows that only a few cortical astrocytes arise from these electroporated SVZ progenitors (Ge et al., 2012). Nonetheless, further postnatal electroporation and retroviral injection experiments show that postnatal SVZ progenitors can produce cortical astrocytes, even to a lesser extent compared to other postnatal source of astrogliogenesis (Ge et al., 2012; Wang et al., 2013; Stogsdill et al., 2017). This result was confirmed with different strategies based on the electroporation of integrative reporters (Clavreul et al., 2019; Figueres-Oñate et al., 2019), including one that showed that pial astrocytes, in addition to protoplasmic astrocytes, are also issued from rapidly dividing SVZ progenitors (Clavreul et al., 2019). Several studies have highlighted the importance of using integrative vs. episomal vectors which are diluted in highly proliferative cells and, therefore, may not recapitulate the whole progeny issued from labeled parent cells (Figueres-Oñate et al., 2015; Clavreul et al., 2019). The heterogeneity and positional identity of VZ/SVZ progenitors that differentially contribute to cortical astrocyte generation may contribute to the cortical astrocyte diversity. For instance, HOPX is a marker of a subpopulation of pallial neural progenitor cells, enriched at the dorso-medial subdomain of the postnatal SVZ that are set to become fibrous astrocyte in the corpus callosum (Zweifel et al., 2018). Finally, fate mapping of Gli1 + progenitor cells in the mouse postnatal cortex revealed a Gli1 + subpopulation of astrocyte progenitors in the SVZ which will eventually generate half of the total cortical astrocyte population (Gingrich et al., 2022). Gli1 being a transcriptional target of Shh signaling, these results indicate that a subpopulation of neonatal progenitors generating cortical astrocytes is defined by Shh signaling and that diversity of astrocyte lineages might contribute to their functional diversity.

#### Postnatal local proliferation

After birth, a major source of protoplasmic astrocytes is the local proliferation of pioneer astrocytes that settle in the cortex (Figure 1). In 1913, Ramón y Cajal first hypothesized that mature astrocytes could divide in the cortical parenchyma. He observed and drew astrocyte doublets connected by their somas. Mitotic figures of astrocytes and/or glia-like cells were later labeled after incorporating the BrdU analogue 3H-thymidine and observed with electron microscopy or with light microscope autoradiography (Fan and Agid, 2018). Later studies showed that local proliferation is already a source of astrocytes at embryonic stages using time-lapse imaging on E18 mouse brain slices in culture (Burns et al., 2009). In this experiment, glial cells expressing GFP after tamoxifen induction in E16 Nestin-CreER;EGFP mice undergo symmetric divisions in the cortical parenchyma every 12 h. The number of glial cells increases significantly during the first postnatal weeks (Bandeira et al., 2009) and local proliferation is a major source of astrocytes in the mouse cerebral cortex at these postnatal stages *via* symmetric divisions of differentiated astrocytes (Ge et al., 2012). The authors showed that 19% of cortical astrocytes are proliferating at P3, and only 1.5% are still dividing at P14. At least in the outer cortical layers, these proliferating astrocytes contribute to nearly half of the astrocyte population primarily through symmetric division. Unexpectedly, dividing parent astrocytes are already differentiated cells exhibiting electrophysiological properties, slightly distinct from the non-dividing astrocytes. Daughter astrocytes functionally incorporate the existing glial network by forming, for example, late perivascular end-feet at P20 (Ge et al., 2012). Strikingly, daughter astrocytes can spread away from their siblings and intermingle with neighboring non-related astrocytes (Clavreul et al., 2019). The proliferative phase is however brief as it essentially occurs before P10 to progressively decline by the end of the third postnatal week in the rat cortex (Moroni et al., 2018).

## Astrogliogenesis

### Astrocyte generation mechanisms at population and individual cell levels

#### Gliogenesis switch

Studying distinct stages of astrocyte development has so far been challenging due to similar markers between astrocyte and neural progenitors together with the lack of stage-specific markers of astrocyte lineage progression. Cortical astrocytes are generated from astrocyte precursor cells (APC), whose molecular identity was so far unknown. APC are generated in at least two temporally distinct waves, either directly or indirectly, from RGC in the developing cortex (Tabata, 2015). After neurogenesis, the first embryonic source of APC arises from the transformation of some translocating RGC. Around birth, the second and principal wave of APC production comes from basal multipotent intermediate progenitor cells that differentiate from their apical analogues previously generated by RGC (Li et al., 2021). Interestingly, both translocating RGC and basal multipotent intermediate progenitors share a common hallmark through the expression of *Ascl1*, *Egfr*, and *Olig2* (Li et al., 2021). In addition, at least some if not all basal multipotent intermediate precursors express Gsx2 after induction by the morphogen Shh that blocks GliR3 (Zhang et al., 2020). EGFR-positive progenitor cells have been also detected in the developing human cortex at the gliogenic switch (Fu et al., 2021). Two subgroups of EGFR + cells, called OAPC and APC, share molecular features with astrocytes and are mainly localized in the outer SVZ. OAPCs express part of astrocyte (SLC1A3, SPARCL1), oligodendrocyte (OLIG2), and outer RGC (HOPX) while APCs express a separate set of astrocyte (SLC1A3, ALDOC) and proliferative (MKI67) marker gene suggesting an immature stage (Fu et al., 2021). After several rounds of proliferation, these multipotent intermediate precursors generate cortical astrocytes and oligodendrocytes as well as a subset of olfactory bulb interneurons at least in mice (Zhang et al., 2020; Li et al., 2021).

#### Astrocyte clone size and composition

Astrocyte clones are highly heterogeneous in terms of size. E13 to E15 progenitors generate astrocyte clones of an average of 8–10 cells, with a high variability up to 40–50 cells (Clavreul et al., 2019; Ojalvo-Sanz and López-Mascaraque, 2021). This maximum size of astrocyte clones is low in the lower layers of the cerebral cortex, compared to its size toward the upper part of the cortex. In terms of astrocyte subtypes, the multicolor method StarTrack, with a GFAP promoter, showed subtype restricted clones, comprised of either protoplasmic or pial astrocytes (García-Marqués and López-Mascaraque, 2013). However, MAGIC markers strategy (Loulier et al., 2014) relying on the ubiquitous CAG promoter, unraveled the bipotency of cortical progenitors with more than 80% of pial astrocytes belonging to heterogenous clones. These heterogeneous clones included both astrocyte subtypes and astrocytes displaying intermediate morphologies encompassing the specific morphological characteristics of the pial and protoplasmic subtypes (Clavreul et al., 2019). Other multicolor clonal analysis of GFAP + cortical progenitors revealed a minority of clones containing sibling cells belonging to both astrocyte and oligodendrocyte lineage (Ojalvo-Sanz and López-Mascaraque, 2021). Therefore, some progenitors maintain the potential to generate different glial cell types. Thus, cortical progenitors are a heterogeneous cell population with respect to which astrocyte subtype they produce, as well as the clonal size and the dispersion of their cell descent. At the clonal level, cortical astrocyte network development appears non-stereotyped. This suggests that the establishment of this network is based on plastic clonal units generated by astrocyte progenitors. These progenitors appear unspecified and capable of expanding and maturing heterogeneously, with their daughter cells probably acquiring their final characteristics through interactions with their cellular and molecular environment. Clonal analysis associated with molecular profiling of astrocyte sister cells should help to better understand the astroglial potential of cortical progenitors in the near future.

### Molecular actors of astrogliogenesis

Among the numerous transcription factors involved in astrocyte generation described in the past years, such as Sox9 and NFIA (Adnani et al., 2018), Zbtb20 is a zinc finger and BTB domain-containing protein 20 transcription factor expressed by neural progenitor cells concomitantly to other family members during the astrogliogenesis phase (Nagao et al., 2016; Medeiros de Araújo et al., 2021). Cortical astrogenesis has been shown to be respectively promoted and reduced by the overexpression and knockdown of Zbtb20 after dorsal electroporation at E15. Astrogenesis is partly promoted by the cooperation between Zbtb20 and NFIA to inhibit the *Brn2* gene involved in neurogenesis (Nagao et al., 2016). The postnatal role of the Zbtb20 has been recently clarified (Medeiros de Araújo et al., 2021). Early conditional deletion of Zbtb20 leads to an increase in a particular subtype of GFAP + astrocytes across all cortical layers. The overexpression of a dominant-negative form of Zbtb20 associated with Primrose syndrome disrupts severely astrogenesis suggesting redundant function between Zbtb family members in astrocyte formation (Medeiros de Araújo et al., 2021). Ezh2 is a histone methyltransferase of the polycomb repressive complex 2 (PRC2) that maintains a transcriptional repressive state in cortical progenitors by methylating the histone H3 at the lysine 27 three times (H3K27me3) (Pereira et al., 2010). This polycomb epigenetic system controls the temporal narrowness of the neurogenic phase in dorsal progenitors and therefore their neural differentiated identity fate. After loss of the PRC2 function in Ezh2-null mice, the developmental timing is accelerated and premature differentiated astrocytes, defined by their GFAP immunoreactivity, are found in the cortical plate from E16 (Pereira et al., 2010). The transforming growth factor-β1 (TGF-β1) is a cytokine that induces premature astrogenesis in the dorsomedial cortex by affecting the polarity of a RGC subset (Stipursky et al., 2014). Released after cortical injuries, blood-derived fibrinogen triggers the differentiation of SVZ neural precursor cells into reactive astrocytes contributing to the scar formation *via* BMP receptor signaling (Pous et al., 2020).

Using comprehensive and integrated transcriptomic and epigenomic analyses to delineate gene regulatory programs from mouse embryonic stem cells toward astrocytes, Tiwari and colleagues pointed out astrocyte-specific genes that acquire priming only upon commitment to the astrocyte lineage and uncovered that epigenetic priming in regulatory elements precedes the stage-specific acquisition of active chromatin and transcriptional activation during astrogliogenesis (Tiwari et al., 2018). They showed *in vitro* that Nfia, Atf3, and Runx2 mediate gene expression programs underlying astrogliogenesis while Nfia and Atf3 promoted astrogliogenesis by suppressing neurogenesis and promoting cell-cycle exit of progenitors, respectively. In addition they demonstrated *in vivo* that Nfia, Atf3, and Runx2 overexpression using *in utero* electroporation of plasmid vectors at E15 steered neurogenic RGC away from generating neurons and promoted astrocyte generation at E18 (Tiwari et al., 2018).

## Astrocyte maturation

The transition from astrocyte progenitor cell to mature astrocyte comes with drastic changes in their morphology (Stogsdill et al., 2017; Clavreul et al., 2019) and gene expression (Cahoy et al., 2008; Zhang et al., 2016).

### Establishment of astrocyte spatial organization at cell and population levels

#### Morphological changes at the cellular level

Cortical astrocytes contact neuronal cell bodies, dendritic spines, nodes of Ranvier, blood vessels, and synapses within their arborization domain (Serwanski et al., 2017; Cohen-Salmon et al., 2021). Cajal already observed a complex arborization using Golgi’s method (Ramón y Cajal, 1909; García-Marín et al., 2007). Recent works relying on endogenous sparse labeling techniques (Hösli et al., 2022) and digital reconstructions (Zisis et al., 2021) have revealed the complex three-dimensional structural details of astroglia processes at the vascular but also at the synaptic interfaces (Torres-Ceja and Olsen, 2022). Astrocyte arborization has been underestimated for a long time due to the lack of immunomarkers labeling not only main processes, as shown with GFAP, S100β, or Aldh1l1 staining, but also fine branches. Expression of fluorescent protein reporters using viral injections, transgenic mouse lines or plasmid electroporations, under the control of promoters, such as gfaABC1D (Stogsdill et al., 2017), GFAP (Halassa et al., 2007), Aldh1l1 (Cahoy et al., 2008), and S100β (Tong et al., 2013), made the visualization of both cell body and complex arborization possible. Expression of a GFAP-GFP reporter in mouse confirmed the existence of astrocyte territorial domains (Halassa et al., 2007; Stogsdill et al., 2017) and multicolor (Livet et al., 2007) or bicolor (Oberheim et al., 2008) lineage tracing studies revealed the territorial organization of astrocyte domains in the rodent cerebral cortex. From P7 to P21, the complexity of cortical astrocyte arborization increases during development (Clavreul et al., 2019) and is concomitant with synaptogenesis and functions (Stogsdill et al., 2017). After the first postnatal week and a phase of proliferation and dispersion, astrocytes undergo a maturation phase where volume and morphological complexity keep increasing at the single cell level. Morphological differences such as cell orientation and arborization complexity are also found between cortical layers (Lanjakornsiripan et al., 2018; Abdeladim et al., 2019). This is particularly true between cortical astrocytes from layers II/III vs. layer VI. Layers II/III astrocytes are more vertically elongated, toward the pial surface while those from layer VI, where neuron morphology and synaptic/dendritic density differ, are more horizontally elongated and less complex (Lanjakornsiripan et al., 2018).

#### Dispersion and organization at the clonal level

With the radial unit hypothesis, Pasko Rakic proposed that the cerebral cortex develops as a cortical columns array, or “radial units,” each originating from distinct RGC located in the VZ (Rakic, 1988). Glial progenitors migrate, similarly to neurons, along radial glia processes (Zerlin et al., 1995). Several strategies combining RGC monocolor sparse labeling and clonal analysis show that their astroglial descent form radial columns (Magavi et al., 2012; Tsai et al., 2012). Cortical columns are composed of both pyramidal neurons and astrocytes (Magavi et al., 2012; Gao et al., 2014). However, multicolor clonal analysis of astrocyte dispersion unraveled a so far underestimated highly heterogeneous dispersion in the mediolateral and anteroposterior axis (Clavreul et al., 2019; Ojalvo-Sanz and López-Mascaraque, 2021). Sibling astrocytes can be found sparsely distributed or forming columns in lower layers or in both lower and upper layers of the cerebral cortex. Astrocyte columns are formed by several disconnected groups or clusters of several siblings. Altogether, these data suggest a discontinuity of the astrocyte network at early stages, with a dispersion of the newly generated astrocytes from embryonic and postnatal progenitors, followed by local proliferation, resulting in intermixed neighboring clones. After this dynamic phase of proliferation and dispersion during the first postnatal week, the cortical astrocyte network organization and dispersion progressively settle down (Clavreul et al., 2019).

### Molecular actors of astrocyte maturation

Astrocytes share a common molecular profile which includes the expression of *Aqp4*, *Dbx2*, *Sox9*, or even *Slc1a3* genes respectively involved in water transport, neural patterning, astrocyte specification, and glutamate uptake (Zeisel et al., 2018; Batiuk et al., 2020). Yet, several transcriptomically different subtypes of mature cortical astrocytes (ACTE1 and 2, AST1 to 3) have been identified in adults due to their unique molecular signature. Two subgroups of telencephalon astrocytes (ACTE1 and 2) have been initially described based on the expression of *Mfge8* and *Lhx2* genes, additionally split into protoplasmic and fibrous/pial astrocytes according to their differential expression of *Gfap* (Zeisel et al., 2018). The diversity of cortical astrocytes has been further examined by combining single-cell RNA sequencing and spatial mapping (Batiuk et al., 2020). Representing 36.5% of ASCA-2 + selected astrocytes, AST1 cells are astrocytes found in the pial layer of the cortex and expressing high levels of *Gfap* and *Agt* (Batiuk et al., 2020). AST2 subtype is evenly distributed between mid-cortical layers and expresses *Unc13c*. AST3 astrocytes are uniformly dispersed throughout the cortex but prevailed in the cortical layer VI and are distinct from the AST1 subtype because they do not express *Gfap*. Using a combination of reporter mice, RNA microarray and histological analyses, another 8.3 astroglia subset has been shown to be enriched in the cortical layer V and expresses GLT1 and LGR6 (Miller et al., 2019). The shared expression of Norrin, modulating local dendritic spines development, by AST 2, 3 and 8.3 subpopulations suggests that the 8.3 astroglia might be included in the AST 2 and 3 subgroups (Batiuk et al., 2020). Interestingly, both AST2 and AST3 cells are two types of non-laminar astrocytes respectively enriched in transcripts linked to glutamatergic and GABAergic neurotransmission suggesting the fine tuning of synaptic function across the different layers of the cortex (Batiuk et al., 2020; Bayraktar et al., 2020). In addition to non-laminar subtypes, some astrocyte subtypes define a new laminar organization of the cortex by expressing layer-specific genes (Bayraktar et al., 2020). Evident and stable from P14, this organization is divided into superficial, mid, and deep astrocyte laminae and differs from the six classical neuronal cortical layers. For example, *Chrdl1* is expressed by upper-layer astrocytes localized in neuronal layers II–IV, while *Il33* is enriched in deep-layer astrocytes in layers V–VI (Bayraktar et al., 2020). Moreover, the laminar astrocyte organization is specific to each cortical area and neuronal cues play an instructive role in the establishment of these laminar astrocytes (Bayraktar et al., 2020). Several subset of astrocytes express neuroactive genes such as *Chrdl1* in upper astrocytes (Bayraktar et al., 2020), *Norrin* in 8.3 astroglia (Miller et al., 2019), AST2 and AST3 astrocytes (Batiuk et al., 2020), or *Sparc/Sparcl1* in subpallial-derived astrocytes (Liu et al., 2022) that modulates synapse formation.

At the epigenetic level, a wide range of transcriptionally active open chromatin is shared between GFAP + cortical astrocytes and Bergman glia (Welle et al., 2021). Binding sites of the nuclear factor I (Nfi) family, known to promote astrocyte differentiation, are enriched in about 25% in these open chromatin regions. Cortical astrocytes execute well specific transcriptional programs centralized around Lhx2 and FoxG1 that are epigenetically controlled. Even in the young adult mouse, astrocytes keep epigenetic marks from their region-restricted RGC specification (Welle et al., 2021). By modeling astrogliogenesis from mouse stem cell coupled to next-generation sequencing and computational approaches, Tiwari and colleagues described regulatory elements and transcriptional programs underlying astrocyte generation and maturation as well as stage- and lineage-specific transcriptomic and epigenetic signatures. More specifically, they demonstrated that Runx2 counteracts action of a reactive phenotype to promote astrocyte maturation *in vitro* (Tiwari et al., 2018). More recently, investigating changes in chromatin accessibility using transposase accessible chromatin using sequencing (ATAC-Seq) in astrocytes isolated from P4 and at 2-month old mouse cerebral cortex, Lattke and colleagues showed that ETS, HOX, ROR families directed chromatin remodeling event and contributed to transcriptional changes associated with astrocyte maturation. In addition, they showed that *in vitro* differentiation of NSC into astrocytes failed to recapitulate *in vivo* maturation as *in vitro* differentiated astrocytes failed to gain chromatin accessibility at many regulatory elements associated with mature astrocyte specific genes (Lattke et al., 2021). Finally, using 3D culture, Lattke and colleagues showed that extrinsic signals, such as FGF2, promoted the transcriptional and epigenetic maturation of astrocytes by making accessible the specific gene sites allowing the maturation of astrocytes. Interestingly, these sites are accessible in adult cortical astrocytes *in vivo*, but not in culture, highlighting the necessity to have the right combination of extrinsic signals and 3D environment to obtain fully matured cortical astrocytes.

## Discussion

All this work in the mouse model sheds light on the complexity of astroglial development in the mammalian brain. This complexity is expected to be even greater in the human brain as many key morphologic and molecular features between rodent and primate/human cortical astrogliogenesis have been highlighted (Majo et al., 2020). For example, two additional categories of astrocytes are found specifically in the human cerebral cortex and absent in the rodent brain: the interlaminar astrocytes in layer I and varicose projection astrocytes in deep layers V–VI. However, the functional significance of this diversity remains elusive yet and the means to investigate *in vivo* these questions out of reach as human cortical astrogliogenesis time frame greatly overlaps with neurogenesis and occurs *in utero* to a large extent (Malik et al., 2013). Despite this challenge, recent progresses have been made in our understanding of the generation of cortical astrocyte diversity in the human brain. By combining analysis of published human cortical single-cell RNA-Seq datasets with immunostainings performed on human fetal brain samples collected around mid-gestation, Yang and collaborators showed that cortical astrocytes, along with oligodendrocytes and olfactory bulb interneurons, were born from basal multipotent intermediate progenitors (bMIPCs) expressing EGFR, ASCL1, OLIG2, and OLIG1 (Yang et al., 2022). Interestingly these bMIPCs are also found in the mouse brain (Li et al., 2021) and thus seems to be a common feature of cortical astrocyte development from these two species. More recently, a distinctive feature between mouse and human cortical astrogliogenesis has been uncovered by Allen and collaborators. By performing fate mapping of VZ and OSVZ niches using local delivery of GFP-expressing viral vectors on organotypic slices of primary human neocortex from gestational weeks 18–23, they showed that astroglial outputs from these two niches were different. While OSVZ progenitor cells generate white matter astrocytes, VZ progenitors give rise to more superficial grey matter astrocytes. This study provides a very good example of the link between the origin and diversity generation of distinct cortical astrocyte subtypes at the morphological and molecular levels in the human brain (Allen et al., 2022). Understanding the details of astrocyte diversity is all the more important as the involvement of astrocytes in human neurodevelopmental disorders is increasingly well-documented. For instance, analysis of the cell type-specific transcriptomic changes in the cerebral cortex of autism spectrum disorder (ASD) patients show early defects in the cellular state of microglia and protoplasmic astrocytes in addition to the disruption of the synaptic signaling of the upper-layer cortical circuitry (Velmeshev et al., 2019). A deeper investigation of alterations of early key steps of human astrocyte development is now within reach thanks to human iPSC-derived 3D cortical spheroids (Sloan et al., 2017). In this powerful *in vitro* model for human astrocyte development, distinct transcriptional profiles between early- and late-stage organoid-derived astrocytes, resembling to primary human fetal astrocytes and mature astrocytes respectively, can be found and recapitulate astrocyte maturation during *in vitro* differentiation. While more data will be needed to characterize the level of astrocyte morphological complexity and glial reactivity in these *in vitro* models, this represents an extremely promising methodology to efficiently model the development of human cortical astrocytes in physiological and pathological contexts. In addition to new models recapitulating astrocyte development, especially in human tissue, a comprehensive analysis of astrocyte morphology, localization, functions, and markers will be needed to better appreciate astrocyte diversity. The first comprehensive and systematic comparison of two regionally distinct astrocytes was recently made possible using a combination of integrated methods encompassing anatomy, electrophysiology, imaging techniques, transcriptomic, and proteomic (Chai et al., 2017). Striatal and hippocampal astrocytes have been shown to be functionally, morphologically, and molecularly distinct, suggesting the existence of neural circuit-specialized astrocytes (Chai et al., 2017). This approach has not yet been used for cortical astrocytes but will help to understand the relationship between astrocyte diversity and function, an essential element to fully apprehend the contribution of astrocyte diversity and its proper generation to a functional brain. Although key features of cortical astrogliogenesis in rodents and primates/humans are progressively unraveled, the complexity of the generation of astrocyte diversity still keeps many of its secrets. Many challenges remain ahead of us. In particular, it will be critical to fully apprehend the similarities and differences between the generation of brain astrocytes in rodents and humans in order to determine the extent to which invaluable mouse genetic models for exploring neurodevelopmental pathologies *in vivo* can be exploited to elucidate the contribution of defective astrogliogenesis or aberrant astrocyte function to these pathologies. Human-specific features, such as the presence of distinctive astrocyte subtypes not found in other species or dedicated progenitors responsible for the generation of particular cortical astrocyte subtypes, remain to also be further challenged in dedicated models such as 3D organoids and in other species to understand how the complex choreography of cerebral cortex development can proceed smoothly despite the specificities of the generation of each distinct cell type. All these aspects will be key to unravel the yet poorly understood causes and course for neurodevelopmental disorders.

## Author contributions

LD designed and realized the figure with the outputs of SC and KL. All authors had the idea for the article, performed the literature search and data analysis, drafted and critically revised the manuscript, and approved the submitted version.
